# Taking the chance: Core self-evaluations predict relative gain in job resources following turnover

**DOI:** 10.1186/s40064-016-3365-0

**Published:** 2016-10-03

**Authors:** Achim Elfering, Anita C. Keller, Martial Berset, Laurenz L. Meier, Simone Grebner, Wolfgang Kälin, Françoise Monnerat, Franziska Tschan, Norbert K. Semmer

**Affiliations:** 1 Department of Psychology, University of Bern, Fabrikstrasse 8, 3012 Bern, Switzerland; 2 National Centre of Competence in Research, Affective Sciences, University of Geneva, CISA, Geneva, Switzerland; 3 Groupe de Psychologie Appliqué, Université de Neuchâtel, Neuchâtel, Switzerland; 4 FHNW School of Applied Psychology, University of Applied Sciences and Arts Northwestern Switzerland FHNW, Basel, Switzerland; 5 Department of Psychology, University of Neuchâtel, Neuchâtel, Switzerland

**Keywords:** Core self-evaluations, Job satisfaction, Job resources, Turnover

## Abstract

Core self-evaluations (CSE) might account for relative gains in job resources across time, especially in situations when these individual differences affect behavior that is relevant for development of job resources. This longitudinal study tests CSE as an individual resource that predicts relative gain in job resources and job satisfaction among job beginners who change or stay with their employer. A questionnaire was filled in by 513 adolescents shortly before the end of vocational training and one year later. Our results replicate previous findings suggesting that job satisfaction is affected by CSE directly and indirectly through the perception of job resources. Multi-group structural equation analysis showed that only leavers had a longitudinal indirect effect of CSE on job satisfaction at the end of vocational training via job resources during their first year of employment. Our findings imply that turnover includes opportunities to optimize one’s circumstances and that CSE helps to attain resourceful jobs.

## Background

As evidenced by a plethora of published studies in the area, job satisfaction is influenced by both situational and dispositional aspects (Arvey et al. 1991; Dormann et al. 2006; Keller and Semmer 2013). Among the most important situational aspects are job control and complexity (Humphrey et al. 2007), and many research findings have shown that personal resources such as positive core self-evaluations (CSE) were consistently associated with higher levels of job satisfaction (Judge and Bono 2001; Stumpp et al. 2009; Wu and Griffin 2012). CSE are basic conclusions or bottom-line evaluations that a person draws about herself or himself (Judge et al. 2005). Individuals high in CSE are expected to be more confident to cope successfully with work tasks and may be less likely to withdraw from complex jobs if they experience failure, because they better believe in their abilities (Judge et al. 2000). In addition, they tend to prefer work tasks that include public speech or negotiating, and they anticipate to be able to cope effectively with difficult situations, while individuals low in CSE may hesitate to perform new tasks. Hence, CSE may be linked to behavior in the workplace that makes it more likely for employees to gain in job resources. The present longitudinal study aims to study the interplay of CSE and job-related resources in a situation of considerable opportunities, that is, the transition from vocational training to work (Elfering et al. 2000).

### Job resources

Work characteristics in general are crucial for employees’ well-being and motivation (Humphrey et al. 2007; Sonnentag and Frese 2013). Often, theories differentiate between job demands and job resources (e.g., Demerouti et al. 2001). Job resources are aspects of the work environment that help dealing with demanding aspects (e.g., time pressure), are functional in achieving work goals, and offer opportunities for learning and personal development (e.g., Demerouti et al. 2001; Hackman and Oldham 1980). Having resourceful jobs enables employees to exert control and successfully master new tasks and challenges, experiences that allow for continuous learning (Bandura 1997; Demerouti et al. 2001; Hackman and Oldham 1980).

It seems intuitive that at the beginning of one’s career, having but also gaining more resources is crucial, as they may facilitate positive career development for several reasons: First, having a resourceful job may allow employees to perform certain tasks when they feel fit for it. This may result in better performance that leads to recognition and rewards over time. Second, job newcomers need to establish themselves in their jobs and are in between exploration and establishment. In both stages, exploration as well as establishment, skill development and learning are crucial developmental tasks (Super 1990), learning processes that can be enabled through job resources. For example, De Witte et al. (2007) found that if job control was high, young employees reported acquiring higher levels of skills in their jobs. Individuals with better skills may be given more interesting tasks and more responsibilities because supervisors assign new tasks to them when they continue to work for the same employer. Third, because job resources tend to offer opportunities for development, they are also associated with more engagement and career competencies such as networking or exploration (Akkermans et al. 2013). In sum, job resources may enable young employees to advance in their careers (Fried et al. 2007). However, people with more personal resources tend to seek challenging and complex jobs (e.g., Best et al. 2005; Srivastava et al. 2010), therefore CSE may facilitate a relative gain in job resources in relation to the mean level, especially when an individual changes his or her employer.

### Core self-evaluations and job resources

Judge et al. (1997) constructed CSE based on self-esteem, generalized self-efficacy, locus of control, and neuroticism. CSE was shown to affect job satisfaction directly and indirectly via the perception of job characteristics (Chang et al. 2012; Judge et al. 2000; Stumpp et al. 2009).

Employees with higher levels of CSE seek resourceful jobs (Best et al. 2005; Judge et al. 2000). Judge and colleagues argued that individuals may differ in on-the-job actions, in that those with higher CSE levels take action to make their jobs more rewarding (Judge and Hurst 2007; Judge and Kammeyer-Mueller 2011). Individuals with higher CSE levels may also perform better at complex tasks because they cope more adequately (Luria and Torjman 2009), believe better in their abilities, and are less likely to withdraw from complex jobs if they experience failure (Judge et al. 2000; Srivastava et al. 2010). If work characteristics do not reflect the environment employees seek, they may engage in behavior to actively change their conditions (Tims and Bakker 2010; Wrzesniewski and Dutton 2001) or leave the organization (Griffeth et al. 2000; Semmer and Schallberger 1996) to achieve a better fit and thus higher levels of job satisfaction. A recent meta-analysis showed that individuals high in CSE tend to report higher levels of job satisfaction and seem to pay more attention to positive aspects of their work environment, resulting in more perceived job resources (Chang et al. 2012).

#### **Hypothesis 1**

At the end of vocational training, CSE have (a) a positive relationship with job resources and (b) a positive indirect relationship with job satisfaction via job resources.

## CSE and staying with one’s employer

In general, gaining more job resources can be the goal of on-the-job actions like job crafting (Tims and Bakker 2010). Job crafting refers to proactive, self-initiated behavior to change work characteristics that are not part of formalized arrangements (Wrzesniewski and Dutton 2001). One implements such self-initiated behavior with the aim of improving one’s work environment so that it fits better with one’s individual abilities, needs, and preferences (Tims and Bakker 2010; Wrzesniewski and Dutton 2001). Employees with higher levels of CSE tend to seek more complex and autonomous work and may be more likely to engage in on-the-job actions to craft their jobs according to their preferences (Judge and Hurst 2007, 2008; Judge and Kammeyer-Mueller 2011). After completing training, it is likely that employees also gain in job resources as a consequence of completion of their training. Such role transitions may be smoother for individuals with more positive self-evaluations. For example, a study showed that young adults with higher levels of self-efficacy reported higher levels of job satisfaction after transitioning from school to work (Pinquart et al. 2003). Therefore we expect adolescents who stay with their organization to be able to craft their conditions for the better—that is, to continuously gain in job resources (crafting effect).

### **Hypothesis 2**

Adolescents who stay with their organization after finishing vocational training (stayer) show a positive longitudinal relation between CSE at the end of vocational training and job resources after completion of vocational training.

## CSE and change of employer

Individuals with higher levels of CSE tend to apply for more complex jobs (self-selection; Srivastava et al. 2010) and may present themselves as competent, capable, and motivated during interviews. Such behaviors may increase their chances of getting hired (selection; Judge et al. 1997; Locke et al. 1996; Pinquart et al. 2003). People with higher levels of CSE tend to choose their job with respect to job characteristics that might fulfill their need for job complexity (self-selection), and organizations try to select individuals for complex jobs who fit into their organizational culture (selection). For example, people with high self-esteem may have more favorable social networks and make better impressions on others; both factors may support successful attainment of challenging jobs (Locke et al. 1996). As a consequence of selection and self-selection, individuals with high levels of CSE are more likely to find themselves in resourceful jobs, which in turn increase job satisfaction (Cohrs et al. 2006; Hackman and Oldham 1980; Humphrey et al. 2007; Judge et al. 2000). Therefore, we expect a longitudinal effect of CSE on job resources. We expect the longitudinal positive association between CSE and job resources to be larger in adolescents who leave their employer after completion of vocational training (leavers) than those who remain with their employer (stayers), because in leavers processes of self-selection and selection should manifest in this particular path and represent a sudden change.

### **Hypothesis 3**

Turnover acts as a moderator: Adolescents who leave their organization show—compared to stayers—a stronger positive longitudinal relation between CSE at the end of vocational training and job resources after completion of vocational training.

### Transition to work in Switzerland

For several reasons the transition to work seems an especially promising period during which to investigate changes in job resources and the role of CSE in attaining more job resources. Many readers might be unfamiliar with vocational training in Switzerland. Hence, a short description of the contextual setting of the current study is given here. In Switzerland, after compulsory schooling, young adults usually graduate from an upper secondary education. The most popular options are matura schools (comparable with college or high school) and vocational educational training (VET). There are two types of VET: full-time training in vocational schools and the more common dual apprenticeship system, in which individuals receive practical training at work and professional education in vocational schools. In dual apprenticeships, young adults are member of the company (cf. Kälin et al. 2000). The transition to work also implies situational changes. People acquire a new status and, for many, this new status is associated with a change in employer because the training contract has ended. However, this period may include neutral and positive change that is more continuous than disruptive and crisis-like (Arnold, 1997; Elfering et al. 2007; Pinquart et al. 2003). Thus, this period seems to be ideally suited for investigating the relationship between CSE and job resources (cf. De Witte et al. 2007).

## Methods

### Participants

For this research we used data from the Work Experience and Quality of Life in Switzerland: Work, Stress, and Personality Development (ÆQUAS) study, which investigates quality of life with a special emphasis on the working life of young people at the end of their vocational training and the subsequent transition into working life.

At baseline, we collected data from 1394 apprentices representing five occupations in their last year of vocational training, which had lasted between 2 years (salespeople) and 4 years (electronic technicians). The population consisted of young people in the German- and French-speaking parts of Switzerland. Data collection in the first wave took place in vocational schools. Follow-up questionnaires were sent by mail after one year. The response rate was 48.4 %, resulting in 675 participants who responded in the first and second waves. An analysis of bias by dropout showed that nonrespondents were significantly lower in conscientiousness; however, we found no significant differences regarding the variables used in this study.

For this analysis we excluded participants from our sample if they did not indicate whether they stayed with or changed their employer after Vocational Educational Training (VET) (exclusion of 74 participants), if they had more than one VET (exclusion of 36 participants), if they did not pass the final exam of VET (exclusion of 10 participants), or if they were unemployed (exclusion of 1 participant). Furthermore, 43 individuals had missing values in indicator variables of the structural model, so that the final sample size for our analysis was 513 participants (90 electronic technicians, 112 bank clerks, 111 nurses, 87 cooks, and 113 salespeople). Their mean age was 20.3 years (SD = 2.3) in the first wave. Language (56.7 % German, 43.3 % French) and gender (43.5 % male, 56.5 % female) were nearly balanced. After the first wave, more than half of the sample (57 %) changed their organization after completing their apprenticeship.

### Measures

#### CSE

We used self-esteem, self-efficacy, and neuroticism to form CSE as a latent variable. The fourth element, locus of control, was not assessed in the panel study. As locus of control shows weaker convergent and discriminant validity, the inclusion of locus of control was controversial since the beginning of CSE research (Bono and Judge 2003; Johnson et al. 2008; Judge et al. 2002). Recent studies further provide theoretical and empirical support for excluding locus of control as an indicator of CSE (Johnson et al. 2011, 2015, 2016).

Self-esteem was measured using five items of the Rosenberg Self-Esteem Scale (Rosenberg, 1979; e.g., “I feel that I’m a person of worth, at least on an equal level with others”). Answering options ranged from 1 (*strongly disagree*) to 5 (*strongly agree*). Cronbach’s alpha was .70 (first wave) and .75 (second wave). Self-efficacy was assessed with four items by Krampen (1991). Three items were positively scored (e.g., “In difficult or dangerous situations, I always know what to do”), and one item was negatively (“In some situations I don’t know what to do”) scored. Participants answered the items using a 6-point scale ranging from 1 (*strongly disagree*) to 6 (*strongly agree*). Cronbach’s alpha for this scale was .69 in Wave 1 and .75 in Wave 2. Neuroticism was assessed using the German version of the NEO Personality Inventory by Costa and McCrae (1985; German version by Ostendorf 1990). The original 45-item adjective rating list was shortened to 30 items by Schallberger and Venetz (1999). Each scale consists of six bipolar items with each pole ranging from *very* (1 and 6), and *quite* (2, 5), to *rather* (3, 4). Cronbach’s Alpha for this scale was .67 in Wave 1 and .74 in Wave 2.

#### Job satisfaction

Job satisfaction was assessed with a three-item measure (Elfering et al. 2000). The first item was a Kunin item asking “How satisfied are you in general with your work?” (Kunin 1955). This item is widely used to anchor evaluations in measures of overall job satisfaction (Cook et al. 1981). It ranges from 1 (*exceedingly unsatisfied*) to 7 (*exceedingly satisfied*). The remaining two items were based on work by Oegerli (1984), for example “I hope my job situation will always remain as good as it is now.” Answer options ranged from 1 (*never*) to 7 (*always*). The three items yielded internal consistencies between .72 and .86 in several studies (Baillod and Semmer 1994; Semmer et al. 2014). Cronbach’s alpha for the scale was .64 in Wave 1 and .74 in Wave 2.

#### Job resources

Job resources were assessed using the Instrument for Stress-Related Task Analysis (ISTA, Semmer et al. 1999). We included items on qualification requirements, participation in decision-making, and job control for an index of job resources (for a composite measure of task related *job resources* see Frese (1999) for a similar procedure with items from the ISTA). Qualification requirements were assessed with an item asking how much knowledge and skills the participant’s work requires, scored from 1 (*very little*) to 5 (*very much*). Participation in decision-making was assessed by an item asking how much influence people had on decisions that concerned their situation as employees, possible answers being “I have no influence” (1), “I just get informed” (2), “I can make suggestions” (3), “I take part in these decisions” (4), and “I have a large influence on these decisions” (5). Job control was measured using an item asking how much one’s work offered possibilities to decide on things, ranging from 1 (*not at all*) to 5 (*very much*).

### Analytical procedure

We applied structural equation modeling using the software package AMOS 19.0 (Arbuckle 2010). Structural equation models combine a measurement model (modeling of latent (unobserved) variables using several observed indicators) with regression analysis to model relationships between variables of interest (e.g., Byrne 2013; Kline 2010).

We first established measurement invariance for our measurement models. We tested whether measurement invariance of item loadings holds for stayers and leavers and if measurement invariance of item loadings holds over time to assure that the same construct has been measured in stayers and leavers and across the observed time period. For longitudinal analyses, at least weak measurement invariance (i.e., measurement models with equal factor loadings over time; Meredith 1993) needs to be given. This is important because otherwise we cannot rule out that observed differences and change are due to measurement error. We compared two models to ensure measurement invariance. In the first model, the item loading was allowed to vary between stayers and leavers as well as over time (freely estimated factor loadings). In the second model, factor loadings were held equal across stayers and leavers as well as over time (constrained factor loadings). Then, we estimated the structural model for the whole sample, and lastly, we applied multi-group modeling to test differences between stayers and leavers (moderator analysis). We applied a longitudinal regression model in which a latent variable at Time 2 is predicted by its autoregression. With this procedure it is possible to control for stability over time (Finkel 1995; Little et al. 2007).

With regard to model fit we followed Hu and Bentler (1998, 1999), who recommended the Comparative Fit Index (CFI), the Root Mean Square Error of Approximation (RMSEA), and the Standardized Root-Mean-Square Residual (SRMR). They suggested that good fit is indicated by values greater than or equal to .95 for CFI, and less than or equal to .06 for RMSEA and .08 for SRMR.

## Results

### Descriptives

Table 1 shows means and standard deviations for leavers and stayers separately. Only mean job satisfaction in Wave 1 differed significantly between the two groups: Stayers reported significantly higher levels of job satisfaction than leavers. Table 1 also shows correlations between factor scores of CSE, job resources, and job satisfaction, showing that stability of job resources and job satisfaction was higher for stayers than leavers.

**Table 1:** Means, standard deviations, and correlation coefficients between factor scores for CSE, work characteristics, and job satisfaction (n = 221 Stayer, n = 292 Leaver)

	Stayer		Leaver		Independent *t* test			1	2	3	4	5
	Mean	SD	Mean	SD	*t* value	*df*	*p*					
1. T1 CSE	3.87	0.46	3.83	0.50	0.77	511	.44	1	.26***	.23***	.19***	.09
2. T1 job resources	3.54	0.53	3.56	0.60	−0.42	511	.68	.14*	1	.34***	.40***	.13*
3. T2 job resources	3.63	0.58	3.57	0.65	1.02	511	.31	.09	.45***	1	.17**	.43***
4. T1 job satisfaction	4.38	1.00	4.07	1.16	3.11	511	<.01	.15*	.32***	.23***	1	.28***
5 T2 job satisfaction	4.14	1.21	4.21	1.30	−0.62	511	.54	.10	.16*	.49***	.39***	1

Pearson correlations between study variables of stayer in lower diagonal, correlation of leaver in upper diagonal

*** *p* < .001; ** *p* < .01; * *p* < .05

### Measurement models and measurement invariance

First, we estimated measurement models for CSE, job resources, and job satisfaction. Factor loadings for CSE ranged from .57 to .81, for job resources from .36 to .66, and for job satisfaction from .51 to .90 (for Wave 1: χ^2^ = 62.1, *df* = 24, CFI = .95, RMSEA = .05, SRMR = .04; for Wave 2: χ^2^ = 51.6, *df* = 24, CFI = .97, RMSEA = .05, SRMR = .03).

We tested longitudinal measurement invariance for job resources and job satisfaction. For both models, Chi square difference tests revealed measurement models with weak invariance did not worsen model fit (for job resources: Δχ^2^ = 1.7, Δ*df* = 3, *p* > .05; for job satisfaction: Δχ^2^ = 5.2, Δ *df* = 3, *p* > .05) but strong invariance was empirically not justified (for job resources: Δχ^2^ = 13.4, Δ *df* = 3, *p* < .05; for job satisfaction: Δχ^2^ = 33.0, Δ *df* = 3, *p* < .05; cf. Table 2).

**Table 2:** Model fit for longitudinal measurement invariance testing of job resources and job satisfaction

	Chi square	*df*	CFI	RMSEA	SRMR
*Job resources*					
Free estimation	4.3	4	1.00	.01	.02
Weak invariance	6.0	7	1.00	.01	.03
Strong invariance	19.4	10	.98	.04	.04
*Job satisfaction*					
Free estimation	9.3	5	.99	.04	.03
Weak invariance	14.5	8	.98	.04	.03
Strong invariance	47.5	11	.95	.08	.05

Our last step in measurement invariance testing was to check for invariance across the two groups. Model fit was not significantly affected by constraining measurement models to be equal across the two groups (free across groups: χ^2^ = 271.6, *df* = 158, CFI = .94, RMSEA = .04, SRMR = .06; equal measurement models across groups: χ^2^ = 282.0, *df* = 164, TLI = .93, RMSEA = .04, SRMR = .06, Δχ^2^ = 10.4, Δ *df* = 6, *p* > .05).

### CSE, job resources, and job satisfaction for stayers and leavers

**Fig. 1:**
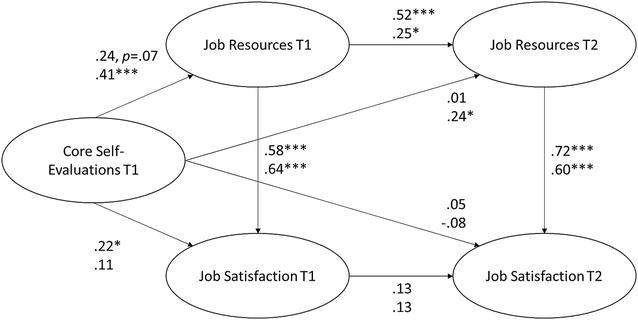
Path diagram illustrating the relationship of core self-evaluations in first wave with job resources and job satisfaction in second wave. Standardized parameters are shown for stayers (upper coefficients, n = 221) and leavers (lower coefficients, n = 292). *p < .05; **p < .01; ***p < .001

To test the longitudinal and concurrent relationship between CSE, job resources, and job satisfaction, we first estimated a model that included all latent variables and regression paths (cf. Fig. 1). This model fitted our data well (χ^2^ = 170.3, *df* = 81, CFI = .95, RMSEA = .05, SRMR = .05). The estimated model for the whole sample showed that job resources in Wave 2 were predicted by job resources in Wave 1 (γ = .38, *p* < .001). The coefficient from job satisfaction in Wave 1 to job satisfaction in Wave 2 was rather low (γ = .12, *p* = .052). CSE in Wave 1 predicted job resources (γ = .34, *p* < .001) and job satisfaction (γ = .18, *p* < .05) in Wave 1. However, CSE in Wave 1 did not predict job resources or job satisfaction in Wave 2. Job resources predicted job satisfaction in both waves (in t1: γ = .58, *p* < .001; in t2: γ = .63, *p* < .001). In partial support of Hypothesis 1, job resources mediated the relationship between CSE and job satisfaction in Wave 1 (γ = .20, *z* = 3.42, *p* > .001, two-tailed; Sobel 1982) but there was no lagged effect on Wave 2.

Next, we estimated a two-group model for stayers and leavers, allowing structural coefficients to vary between groups. Again, this model showed good model fit (χ^2^ = 296.5, *df* = 168, CFI = .93, RMSEA = .04, SRMR = .07). To test whether differences between the two groups were meaningful, we constrained structural coefficients to be equal across the two groups. Goodness-of-fit criteria did drop significantly (χ^2^ = 321.5, *df* = 176, CFI = .92, RMSEA = .04, SRMR = .08, Δχ^2^ = 25, Δ *df* = 8, *p* > .05).

As expected, the stronger positive longitudinal path was in leavers, showing that CSE in Wave 1 predicted job resources in Wave 2 (γ = .24, *p* < .05) even when the stability in job resources and the synchronous effect of CSE on job resources were controlled for (cf. Fig. 1). Notably, the indirect effect of CSE in Wave 1 on job satisfaction in Wave 2, mediated by job resources in Wave 2, was also meaningful (γ = .25, *z* = 2.20, *p* < .05, two-tailed; Sobel 1982). When looking at the total effects of CSE on job satisfaction, the mediation is absent in stayers, but turned out to be 78 % of the total effect in leavers (cf. Table 3). Therefore, Hypothesis 2, stating that stayers will report an increase in job resources, was not supported because the indirect path was not significant in stayers. However, our results were in line with Hypothesis 3.

**Table 3:** Direct, mediated, and total relationships between core self-evaluations and job satisfaction

	Wave 1		Wave 2	
	Stayers	Leavers	Stayers	Leavers
Direct	.22*	.11	.05	−.08
Mediated (all indirect paths)	.14	.26	.14	.25
Mediated by perceived work characteristics	.14	.26**	.00	.14*
Total	.36	.37	.19	.18
Proportion of relationship mediated by perceived work characteristics	.39	.70	.00	.78

Standardized parameter estimates

## Discussion

During the last decade a growing body of evidence has indicated that CSE relates to job satisfaction (Bono and Judge 2003; Dormann et al. 2006). We were able to replicate previous findings that CSE predicted job resources and job satisfaction. Furthermore, the relationship between CSE and job satisfaction was mediated by the perception of job resources. For the whole sample, these effects were only found within one wave but not over time. Thus, the first hypothesis was only partially supported. However, our results were different depending whether adolescents stayed with or changed their organization. For stayers, we found the same results as for the whole sample: Job satisfaction was predicted by job resources and CSE in Wave 1. For leavers, however, we found a positive longitudinal link of CSE, job resources and job satisfaction.

Among young adults who stayed with their organization we found no longitudinal effect from CSE on job resources one year later. We see two possible explanations for this result: First, if after completion of VET young employees stay with their organization, it is likely that they work with the same team as they did before they completed their education. Working with the same employees, adolescents may still be regarded as greenhorns and may not receive opportunities to gain more job resources. This would also imply that the role transition from learner to full employee has not yet been fully made. Second, shortly after transition, adolescents may still receive close supervision and monitoring from supervisors, circumstances which hinder job crafting (Wrzesniewski and Dutton 2001). A study by Berg et al. (2010) indicated that lower-rank employees, a category that young adults right after their VET are likely to belong to, have less autonomy and face difficulties when actively trying to craft their jobs. For example, lower-rank employees may have a lack of power that may make it necessary for them to get the approval of their supervisor (Berg et al. 2010). Even if employees are less satisfied and perceive the need for adapting their work environment, at this stage resources for job crafting may be invested in other areas, such as becoming more independent from one’s (former) supervisor, than in increasing instrumental job resources.

Our study showed that more than half of the sample reported significantly lower job satisfaction and changed employers after their VET. During these self-selection and selection processes many behaviors that relate to individual differences in self-esteem, self-efficacy, and emotional stability (i.e., CSE) are needed, such as searching for an interesting job, writing a persuasive application, communicating goals and needs in interviews for a job, etc. It seems that young adults with higher CSE who leave their employers are able to attain job resources that may be in line with their needs and abilities. Our results seem to offer further evidence that people with high CSE seek more complex work (cf. Judge et al. 2005; Srivastava et al. 2010). Young adults with higher levels of CSE applied for resourceful jobs (self-selection) and may have presented behavior that increases the likelihood of getting hired during selection processes (e.g., confident appearance; selection). Besides these proactive behaviors and better abilities to cope with change, people with higher levels of CSE may be more likely to engage in self-initiated career planning and job exploration as it becomes necessary with modern careers (Arthur et al. 2005; Judge and Kammeyer-Mueller 2011). Such careers involve movement within one organization and across organizations. People with successful careers are characterized as being confident in their abilities, developing goals independently, and taking initiative to develop their own competencies (Hall 2002), characteristics that conform to high CSE (Judge and Kammeyer-Mueller 2011).

The reported relationships in this study may also be linked to recognition, rewards, and successes. Recognition and rewards include strong positive performance feedbacks and are experienced as subjective success. Previous research showed subjective occupational success including four success-subdimensions (i.e., positive feedback, goal attainment, pro-social success, and career success) to buffer the stressor-strain relationship (Grebner et al. 2010). Meanwhile, experiences of success, especially through goal attainment is also known to result in setting of higher goals and acquisition of complex tasks and resources to reach those goals as predicted by the high performance circle (Latham et al. 2005). Hence, future studies should test whether recognition, rewards, and subjective occupational success functions as an enhancing moderator between CSE and job resources or CSE and job satisfaction.

## Limitations

While we do believe that our study has contributed to knowledge of the interplay between personal and job resources, it is not without limitations. The most important is connected to our sample. The response rate of 48 % in Wave 2 is not random, as dropouts were significantly lower in conscientiousness. However, when we compared separate measurement models for the longitudinal sample and the dropouts in Wave 1, a model with equality restraints on indicator loadings and correlations between latent constructs was not noticeably different from a model where these parameters were freely estimated (Δχ^2^ = 15.2, Δ*df* = 9, *p* > .05). Thus, there is not much evidence for bias in construct validity, and support for the concept of CSE remains intact. The study is not fully conclusive regarding the extent to which personality-related behavior contributed to the longitudinal mediation effect. In fact, we lack more specific information on the turnover process, how often individuals applied for what job offers, and what information young workers used to decide to apply for a job. Future studies should collect these data in order to quantify personality-related self-selective strategies and disentangle self-selection and selection effects. Further, the study only captured two time points. CSE may not only predict direction of change in resources and satisfaction over time, but also velocity of these changes. Future studies on CSE and turnover may incorporate more time points to gain a better understanding of the dynamic relationships over time. Finally, one potential drawback of this study is that analyses are based on the assumption that the data and the underlying processes are uniform while in reality contextual moderators like finiteness or decrease of the resources may create competition and dependencies. Future studies may try to model resource limiting conditions and long range dependence in statistical analyses (e.g., Bogdan 2015). While the stability of job resources and job satisfaction was addressed via autoregression in the current study, the history of job resources and job satisfaction and its impact may be more complex over time. In future multi-wave studies it would be interesting to see how new autoregressive models with long range memory developed for multidimensional and multimodal data would fit the data (e.g., Xue et al. 2016).

## Implications and conclusion

Young adults at the end of their apprenticeship may be encouraged to change their employers because this transition may offer opportunities to improve their resources at work. However, young adults with lower levels of self-esteem, self-efficacy, and emotional stability may benefit from mentoring during this period in order to increase their likelihood of attaining work characterized by sufficient resources. For young adults who stay with their organizations, supervisors may offer opportunities to take responsibility and participate in decision-making. Our results also highlight the fact that job satisfaction is in part affected by personal resources such as CSE but also by job resources. Employees whose employers grant them higher autonomy and participation in decision-making, as well as encourage them to acquire new skills, report higher satisfaction levels, which in turn tend to be associated with a variety of desired organizational outcomes such as higher commitment or performance (Humphrey et al. 2007).

In the context of our study, behaviors that are relevant for finding a new job (e.g., searching for and exploring new jobs, interviewing, etc.) may be interpreted as having low avoidance motivation, which means having a decreased sensitivity to perceive harmful information. For example, a job hunter may ignore a required skill he or she does not have (e.g., a second language) in a job posting. Its counterpart, approach motivation (i.e., increased sensitivity to incentives and positive information), may be responsible for setting high goals (e.g., to fulfill complex and challenging work) and thus gaining more resources at work. In our example above, this then would make the job hunter focus on job requirements he or she fulfills or believes he or she is capable of fulfilling in the future. Having this attitude may help in presenting oneself in an appealing light to employers. To date, the CSE concept only includes characteristics that represent avoidance but not approach motivation (e.g., extraversion). During turnover processes, where presentation of oneself and social encounters are crucial, approach motivation may even be a better predictor for success (Johnson et al. 2008).

In sum, development in resources at work (e.g., improving autonomy) is related to high CSE for young adults who change their employer. CSE is an individual resource that assists gains in job resources in situations that afford behavior that is modulated by CSE. The study therefore adds knowledge to cycles of gain between personal and job resources.
